# β-Sitosterol-loaded solid lipid nanoparticles ameliorate complete Freund’s adjuvant-induced arthritis in rats: involvement of NF-кB and HO-1/Nrf-2 pathway

**DOI:** 10.1080/10717544.2020.1818883

**Published:** 2020-09-18

**Authors:** Feng Zhang, Zhiyu Liu, Xijing He, Zhanqi Li, Bin Shi, Fengmei Cai

**Affiliations:** a Department of Orthopedics, The Second Affiliated Hospital of Xi'an Jiaotong University, Xi'an, China; b Department of Orthopedics, Xi'an Fourth Hospital, Xi'an, China; c Department of Pathology, Xi'an Fourth Hospital, Xi'an, China

**Keywords:** β-Sitosterol, nanoparticles, arthritis, inflammation, HO-1/Nrf2 pathway

## Abstract

Rheumatoid arthritis (RA), autoimmune disease that is categorized via chronic inflammation manifestation, obesity, cardiovascular risk and even enhanced the mortality and affect the 0.3 and 1% of population worldwide. The current experimental study was scrutinize the anti-arthritic effect of β-sitosterol loaded solid lipid nanoparticles (SLN) against complete Fruend adjuvant (CFA)-induced arthritis via dual pathway. Double emulsion solvent displacement method was used for the preparation of β-sitosterol solid lipid nanoparticles (SLN). CFA was used to induce arthritis and rats were divided into different groups for 28 days. Biochemical, anti-inflammatory, pro-inflammatory cytokines and inflammatory mediator were estimated, respectively. Receptor activator of nuclear factor kappa-B ligand (RANKL), signal transducer and activator of transcription-3 (STAT3) nuclear factor erythroid 2–related factor 2 (Nrf_2_), Heme Oxygenase-1(HO-1) and Nuclear factor-κB (NF-κB) expression were estimated. β-sitosterol-SLN significantly (*p* < .001) reduced the paw edema, arthritic index and increased the body weight. β-sitosterol-SLN increased the redox status of synovium {reduce the malonaldehyde (MDA) and increase superoxide dismutase (SOD), glutathione (GSH) and catalase (CAT)} level and reduced the cytokines such as tumor necrosis factor-α (TNF-α), interleukin-1β (IL-1β), interleukin-2, interleukin-6, interleukin-16, interleukin-17 and increased level of interleukin-10, Transforming growth factor beta (TGF-β). β-sitosterol-SLN significantly (*p* < .001) reduced the level of cyclooxygenase-2 (COX-2), prostaglandin E_2_ (PGE_2_), vascular Endothelial Growth Factor (VEGF) and NF-κB. β-sitosterol-SLN significantly increased the expression of HO-1,Nrf_2_ and decreased the expression of NF-κB, RANKL, STAT3. In conclusion, β-sitosterol SLN showed the antiarthritic effect via suppression of NF-kB and activation of HO-1/Nrf-2 pathway.

## Introduction

Rheumatoid arthritis (RA), autoimmune disease that is categorized via chronic inflammation manifestation, obesity, cardiovascular risk and even enhanced the mortality and affect the 0.3 and 1% of population worldwide. RA categorized via inflammation, synovial membrane, swelling cartilage destruction, autoantibody production and bone destruction. Research suggest that the RA mostly affect the middle-aged females (50–60 years) and women are most affected as compared to man (Rutherford et al., 2017). RA is also liked with the systemic complications, early mortality, socioeconomic cost and disability. During the expansion of RA, its affects the hands joints and feet resultant in a steady painful swelling, pannus formation, abnormal expansion of synovium, exaggerate and changes in the joint morphology (Funk et al., 2006). The exact initiation mechanism of arthritis is still not fully clear, but some researcher and previous publish report suggest that the various pro-inflammatory cytokines, immune cell populations and inflammatory mediators is admitted. During the pathological process and early symptoms of swelling, heat, reduced joint function and pain; the later stage exhibit different degrees of deformity and joint stiffness accompanying bone damage and disability risk (Woodruff et al., 2002; Funk et al., 2006; Petchi et al., 2015). Research suggests that inflammation is a physiological reaction against harmful stimuli, such as pathogens in the body. By inducing the release of signaling molecules, inflammation has a protective effect that deactivates various deleterious pathogens (Funk et al., 2006; Amresh et al., 2007; Jalalpure et al., 2011).

Currently, no effective treatment is available for the RA, but the clinical and physician used the non-steroidal anti-inflammatory drugs (NSAIDs), disease modifying and glucocorticoids drug for the treatment (Kumar et al., 2015a,b). Conversely, recent advancement, NSAIDS is the choice of drug but these therapies having the limitation due to their side and adverse effects like cardiac toxicity and gastrointestinal effects. Another treatment glucocorticoid showed the side effects like metabolic disorder and osteoporosis. In the recent year, the researcher targeted the pro-inflammatory cytokines and inflammatory mediators to treat the RA and its complication. Previous research suggest that the macrophage play a defence role against the invading agent such as viruses, fungi and bacteria (Kumar et al., 2015a,b, 2016b). Due to the invading agent attack, start the secretion of cellular signaling molecules and numerous pro-inflammatory cytokines such as tumor necrosis factor-α (TNF-α), interferon-γ (IFN-γ), interleukin-6 (IL-6) and interleukin 1-β (IL-1β) and inflammatory mediators like prostaglandin E2 (PGE2), nitric oxide (NO) and cyclooxygenase-2 (COX-2). Moreover, de-regulated cytokine production and induction of inflammation are liked with the conditions viz., diabetes, arthritis, cancer, obesity and cardiovascular disease. Therefore, urgent need of one effective therapeutic agent to treat the arthritis via regulation the production of pro-inflammatory mediators (Kumar et al., 2016a; Rahman et al., 2016, 2017). Published literature suggests that numerous inflammatory cells are present to infiltrate the affected RA inflammatory sites of patients suffering from pro-inflammatory cytokines, such as IL-6, which catalyzes the spread of native CD4+ cells to the T helper of 17 lymphocytes by enhancing signal transducer and transcription activator-3 (STAT-3) and intracellular expression (Sengupta et al., 2011; Kamel et al., 2018).

Pro-inflammatory cytokines, such as IL-17, play a crucial role in the expansion and decline of RA via boosting the synovial fibroblasts and immune cells along with boosting the expression of nuclear factor (NF-κB) and down-regulating the cascade such as inflammatory mediators like TNF-α (Sengupta et al., 2011; Kamel et al., 2018). IL-17 increases the production of reactive oxygen species (ROS). Significantly, pro-inflammatory cytokines like TNF-α, ROS, and IL-17 are functioning synergistically, inducing expression of vascular endothelial growth factor (VEGF). Previous research has reported that VEGF plays a crucial role in the inflammatory response, and that angiogenesis is also the cause of chronic rheumatoid inflammation (Sengupta et al., 2011; Gao et al., 2016; Kamel et al., 2018).

Activity induces synoviocytes to induce elevated levels of toxic cytokines such as kappa-B ligand nuclear factor (RANKL) and matrixmetalloproteinase-3 (MMP-3). RANKL is a crucial enzyme for RA cartilage pathology destruction, as proteoglycs and the form of collagens (IX and X) are digested. MMP-3 is the principal cause of bone degradation in RA and functions as a differentiating osteoclastic cause (Arham et al., 2016; Kamel et al., 2018).

As in the case of various natural compounds, poor aqueous solubility, resulting in low bioavailability and low targeting efficacy, has restricted clinical development of production of β-Sitosterol. Its well documented that nanoparticle drug delivery system increases the therapeutic efficacy of various natural compounds. Several new nano-formulations have been used in a recent study to improve the therapeutic potential of a large number of naturally derived drugs such as umbelliferone, resveratrol, vincristine, curcumin, silymarin rutin, paclitaxel, artemisinin, camptothecin, honokoil and green tea catechins (Bilia et al., 2017). Many of these natural products are already on the market, others are undergoing clinical trials (Bilia et al., 2017).

As my knowledge, few of the studies have been carried out on the β-sitosterol to improve the therapeutic efficacy. Most of the investigations conducted with β-sitosterol have focused on using it and its derivatives as an excipients to regulate drug release or promote drug absorption (Farkas et al., 2006; Lacatusu et al., 2012). In previous study, cyclodextrins has been used to increase the aqueous bioavailability and solubility of β-sitosterol. Imanaka et al., showed that the liposomal containing formulation of β-sitosterol enhanced the natural killer cell activity and reduced the B16BL6 melanoma cells colonies in the lungs of experimental mice (Imanaka et al., 2008). Awad et al., showed the protective effect of β-sitosterol against various cancer cells via involving the 2-hydroxypropyl-β-cyclodextrin (HP-βCD) as a carrier vehicle (Awad et al., 2000, 2001, 2007). These investigations showed that β-sitosterol reduced the cell proliferation after the 3–5 days at 16–32 µM. It is well documented that cyclodestrins commonly used in the pharmaceutical preparations as excipients to increase the bioavailability and solubility. We hypothesized that solid lipid nano-particles of β-sitosterol can offer better enhancement of antiarthritic effect against the rodent model. This assumption is due to various beneficial effect of SLN such as improved drug-loading capacity, controlled release, facilitated, improved solubility and targeted drug release.

Solid lipid nanoparticles (SLNs) are the most promising oral drug delivery carriers due to their long term stability, high drug-loading capacity, excellent biocompatibility, large-scale production viability and long-term stability. Furthermore, previous researcher suggests that the SLN increased the intracellular drug delivery for anti-arthritic drugs (Arora et al., 2015; Ahmad et al., 2018). SLNs are a colloidal system primarily formulated of solid lipids. SLNs get the more popularity against the numerous poorly soluble drugs such as anti-arthritic drug.

With regard to research work carried out in literature to boost β-sitosterol biopharmaceutical efficiency using nanocarriers, the specific formulation such as PLGA-loaded β-sitosterol nanoparticles (Andima et al., 2018). While these discuss formulation exhibited a definite improvement in the drug's biopharmaceutical attributes, the current investigation was undertaken to discuss the significant finding of β-sitosterol-SLNs systematically optimized to enhance the effectiveness of drug therapy against CFA-induced arthritis.

## Material and methods

### Chemical

Complete Fruend’s adjuvant (CFA) was procured from the Chondrex, Inc. (U.S.). Lipopolysaccharides (LPS), indomethacin and β-sitosterol were purchased from the Sigma Chemical Company, St. Louris, MO, U.S.A. IL-1β, IL-6, IL-10, IL-16, IL-17 and TNF-α were purchased from the U-CyTech Biosciences. Rest of the chemical used in the current experimental study were procured from the reputed vendors.

### Preparation of the β-sitosterol-Solid lipid nano-particle of (β-sitosterol-SLNs)

Solvent diffusion and hot homogenization model was used for the fabrication of SLN of β-sitosterol via using the previous reported protocol with some modification. The quantity of solid lipid was used for the formulation of SLN was selected on the solubility basis. 250 mg Compritol 888 ATO, 60 mg Phospholipid 90 G (PL90G) was used for the formulation of solid lipid. Both the co-surfactant and solid lipid were melted upto 70 °C and fixed amount of β-sitosterol (50 mg) were added with continuous stirring for complete soluble in the liquid (lipidic). After that, tween 80 (3% w/v) solutions was separately prepared (soluble in the 10 mL). The aqueous phase was dissolved into the organic phase under the continuous homogenization for 2–6 min at 10000 rpm to obtain the uniform dispersion. Additionally, 10 mL of water added into the solvents and then constantly stirred for 1–4 h at 1600 rpm in ice bath for acquisition of SLNs and finally SLNs kept in the refrigerator for further use.

### Characterization of β-sitosterol-Solid lipid nano-particle of (β-sitosterol-SLNs)

#### Particle size (PS)

Dynamic light dispersion technique (M/s Malvern Instruments, Worcestershire, UK) was used for the determination of particle size distribution of β-sitosterol-SLNs.

#### Drug loading capacity (LC) and entrapment efficiency (EE)

The loading capacity and entrapment efficiency are well known proportion of drug effectively struck in the SLNs. The LC and EE were estimated via using the previous reported method of Rahman et al., with minor modification (Rahman et al., 2019). In brief, 2 mL dispersion aliquot of SLN was ultra-centrifuged and supernatant discarded and collected the pallet. Additionally, pellet comprising SLN was finally applied for ultrasonication for 15 min to complete remove the drug from formulation. Finally, the drug collected in mobile phase and estimated via high-performance liquid chromatography (HPLC). The below given equation was used for the estimation of loading capacity and entrapment efficiency.

$$\mathrm{LC}\;(\%)=\frac{\mathrm{Entrapped}\;\mathrm{Drug}}{\mathrm{Nanoparticles}\;\mathrm{weight}}\times 100$$

$$\mathrm{EE}\%=\frac{\mathrm{Total}\;\mathrm{quantity}\;\mathrm{of}\;\beta -\mathrm{sitosterol}–\mathrm{Amount}\;\mathrm{of}\;\beta -\mathrm{sitosterol}\;\mathrm{in}\;\mathrm{total}\;\mathrm{supernaent}}{\mathrm{Amount}\;\mathrm{of}\;\beta -\mathrm{sitosterol}}\times \;100$$

#### Transmission electron microscopy (TEM)

The 1 mL aliquot of SLNs was 100 time diluted with the triple distilled water and finally distributed on the copper grid with phosphotungstic acid solution (1%), and finally, the prepared sample was observed under the transmission electron microscope (JEM-2100F, M/s Jeol, Tokyo, Japan).

### *In vitro* drug release study

*In vitro* drug release study was carried out to estimate the release pattern of β-sitosterol-SLNs. Briefly, the SLNs dissolved in phosphate buffer saline (PBS; pH-7.4) at 37 °C at 100 rpm for 24 h. finally the SLN was packed in the dialysis bag. 2.5 mg of SLN dispersion was loaded into the dialysis bag and used for the current experimental study. 0.5 mL aliquot of the samples were taken at regular time interval and finally loaded the equal amount of fresh medium at 37 °C. HPLC spectroscopy was used for analyzed the sample and estimation the percent drug release. The received drug release was fitted with different mathematical model includes zero, first, Higuchi and Korsmeyer – Peppas model to scrutinize the release kinetic pattern.

### Stability studies

The stability study was carried out at 25 °C/60% RH and 40 °C/75%RH for estimation the stability of formulation. Briefly, the formulation was kept in the tight-sealed vials and subjected to perform the stability studies at different time interval (0, 1, 2, 4, 8 and 12 weeks). These formulations are scrutinized for different parameter such as PDI, PS, LC and EE at different time intervals.

### Experimental rodent

Swiss Wistar rats (150–190 g, sex – male) used for the current experimental study. All the experimental study was carried out according to the Institutional guidelines. All the experimental rats kept in the standard laboratory condition such as 22 ± 5 °C temperature; 60–75% relative humidity and 12-h dark and light cycle.

### Induction of arthritis via complete freund’s adjuvant (CFA)

Complete Freund’s adjuvant (CFA) was used for the induction of arthritis. 0.1 ml injections of Wistar rats were injecting the CFA in the right hind metatarsal foot pad with equal volume of saline in the rat control group. The day of injection, day 0 immunization.

### Drug administration

Tested and standard drug were suspended into the 1% solution of carboxyl methyl cellulose (CMC) and orally administered to the experimental rats for 28 days. Normal control group rats received the 1% solution of CMC.

### Experimental protocol

The experimental rats were divided into different groups as follows

Group I: Normal control
Group II: CFA received only
Group III: CFA + β-sitosterol (2.5 mg/kg)
Group IV: CFA + β-sitosterol (25 mg/kg)
Group V: CFA + β-sitosterol-SLN
Group VI: CFA + Indomethacin (400µg/kg)

The rats were received the over mention treatment for 28 days. The paw edema, body weight, water and food intake were estimated at regular time intervals. At end of the experimental study, the rats were anesthetized and blood samples were withdrawn via puncturing the retro orbital (Kumar et al., 2015a,c).

### Index of spleen and thymus

At end of the experimental study period, the rats were scarified via using the excess of anesthesia and immediately thymus and spleen tissue was successfully removed from the all group of animals and weighted. For the estimation of thymus and spleen index, the index was presented at a ratio (mg/g) weight versus body weight, respectively.

### Poly-arthritic index

The arthritis index and paw swelling were screened regularly in order to determine the extent of arthritis. Plethysmometer was used for the estimation of paw swelling and the data was presented in the form of paw volume calculated via subtracting the basal volume. Previously used method was used for the estimation of inflammation in the paw via given the score. Score 0: paws have no swelling or may be focal redness; score 1: figure joint persist the swelling; score 2: swelling of the wrist joints or ankle; score 3: expand inflammation of entire paw and score 4: paws ankylosis or deformity. Each paw was calculated separately and the cumulative scores of 4 paws of each rat were used as polyarthritis index with a maximum value of 16 per rat.(Kumar et al., 2015a,c, 2016b).

### Antioxidant parameters

For the biochemical parameter estimation, the tissue was successfully removed from excised ankle joint all experimental group rats weighted and stored at −80 °C. Further, the tissue homogenate (10%) was prepared in phosphate buffer saline and used for the estimation of catalase (CAT), malonaldiadehyde (MDA), superoxide dismutase (SOD) and glutathione (GSH), respectively.

### Hematological parameters

Hematological parameters such as erythrocyte sedimentation rate (ESR), white blood cells (WBC), hemoglobin (Hb) and red blood cells (RBC) were estimated using the previous reported method with minor modification (Jalalpure et al., 2011; Kumar et al., 2016b).

### Hepatic parameters

Hepatic parameters such as Aspartate transaminase (AST), alkaline phosphatase (ALP) and Alanine transaminase (ALT) were estimated using the colometric kits (JOURILABS kit).

### Cytokines estimation

ELISA kits were used for estimating pro-inflammatory cytokines including IL-6, IL-1β, IL-10, Il-16, IL-17 and TNF-α (U-CyTech Biosciences) were estimated following the manufacture instruction. Pro-inflammatory cytokines were estimated in the serum were estimated pg/ml and in tissue was estimated pg/mg.

### Inflammatory mediators

Inflammatory mediators such as NF-κB, COX-2, VEGF and PGE_2_ were estimated using the ELISA kits following the manufacture instruction.

### Quantitative RT-PCR

SV total RNA isolation system was used for separation of total RNA from the high limb tissue of rats (Promega, Madison, WI, USA). The purity of RNA was confirmed via using the spectrophotometer at 260 nm. RT-PCR kit was used for reverse transcribed into cDNA (Stratagene, Santa Clara, CA) using the manufacture instruction. Briefly, QuantiFast SYBR Green PCR master mix (25 μl) mix with primer pair mix (2 μl), dH_2_O (22.5 μl) and cDNA (0.5 μl) and maintain the final volume 50 μl. The sequences of the primer listed in Table 1. PCR included 95 °C for 10 min activate the AmpliTaq DNA polymerase, followed via 40 cycles for 15 s at 95 °C (denaturing) and 1 min at 60 °C (extension/annealing). The results were presented in cycle threshold (C_t_), where the enhanced fluorescence curve passes across a threshold value.

**Table 1.** The list of primer.

S. No	Gene/	Primer	
		Reverse	Forwarded
1	HO-1	CCTCTGGCGAAGAAACTCTGTC	ACCCCACCAAGTTCAAACAGC
2	Nrf-2	TCGGCTGGGACTTGTGTTCAGT	GCCTTCCTCTGCTGCCATTAGT
3	NF-kB	GAGGAAGGCTGTGAACATGAGG	TTCTGGTGCATTCTGACCTTGC
4	RANKL	GAAGGGTTGGACACCTGAATGC	CACACCTCACCATCAATGCTGC
5	STAT-3	TGACCTGCCACCTGACAGTA	GTAGGGGTTCCTCACCCTTC
6	GAPDH	AGACAGCCGCATCTTCTTGT	TGGAAGATGGTGATGGGTTT

### Statistical analysis

All the data provided in the current experimental analysis as means ± Standard Medium Error (SEM). One-way analysis of variance (ANOVA) was used for comparison between the various groups, followed by comparison testing with Dennett. When *p* < .05 was perceived to be substantial difference. GraphPad Prism software was used for the statistical analysis.

## Result

### Validation of optimized model

The prepared β-sitosterol-SLN nanoparticles were found in the spherical shape with uniform in size distribution. The formulated nano-particles optimized via using the various parameters like particle size in size, showing the validly of predicted model. The prepared β-sitosterol-SLN showed the averaged mean percentage, mean loading capacity and entrapment efficiency. Transmission electron microscopy (TEN) exhibited the nano size range and spherical shape of prepared β-sitosterol-SLN (Supplementary figure 1).

### *In vitro* release of β-sitosterol -SLN

*In vitro* permeation experiment was carried out to estimation the permeation of β-sitosterol-SLN and control. invitro permeation profile via excised abdominal rat skin. The β-sitosterol-SLN skin permeation profiles complied with the Fick 's diffusion rule. Statistical analysis for 24 h experimental study, demonstrated the higher flux for β-sitosterol-SLN as compared to control (β-sitosterol control) (Table 2). Whereas the cumulative amount of β-sitosterol permeate from SLN was almost 4 times higher as comparison to control.

**Table 2.** *In vitro* skin permeation studies of β-sitosterol-SLN and control.

Formulation Code	Flux (µg/cm^2^/ hr)	Permeability Co-efficient × 10^−3^(cm/hr)	Enhancement ratio
β-sitosterol -SLN	14.34	1.98	3.98
Control	3.21	0.29	1

### Characterization of β-sitosterol-SLN

#### Drug loading capacity (LC) and entrapment efficiency (EE)

The β-sitosterol-SLN exhibited the high LC (14.1%) and EE (90%) presented in the Supplementary Table 1.

### Stability studies

#### Particle size distribution and polydispersity index (PDI)

The optimized β-sitosterol-SLN particle size stored at 25 °C/60% RH (73.06 to 67) and 40 °C/75% RH (66.01 to 105 nm) presented in the Supplementary Tables 1 and 2. During the storage at 25 °C/60% RH, the particle size of the β-sitosterol was quite unchanged, while at 40 °C/75%RH the particle size of β-sitosterol was enhanced due to aggregation phenomenon. However, Supplementary Table 1 showed the low and steady values, thereby at 25 °C/60% RH, showing the stability of PS for next 12 weeks.

#### Effect of β-sitosterol-SLN on paw edema

Table 3 exhibited the effect of β-sitosterol-SLN on the experimental rats. CFA-induced rats demonstrated the increased paw edema at increase the time and reached maximum at day 21 (3.7 ± 0.69 cm). CFA-induced rats treated with β-sitosterol (2.5 and 25 mg/kg, body weight) exhibited the reduction in the paw edema at the day 28 (2.2 ± 0.89 and 1.4 ± 0.93 cm). β-sitosterol-SLN treated group rats exhibited the maximum diminution in the paw edema (0.43 ± 0.07 cm). A similar result was obtained for the group treated with indomethacin.

**Table 3.** The effect of β-sitosterol on the paw edema of CFA-induced arthritic rats.

S. No	Groups	Increase in Joint diameter (cm) (days)			
		Day 7	Day 14	Day 21	Day 28
1	CFA	2.8 ± 0.43	3.1 ± 0.78	3.7 ± 0.69	3.5 ± 0.72
2	CFA+ β-sitosterol (2.5 mg/kg)	2.1 ± 0.72*	2.5 ± 0.83**	2.4 ± 0.74***	2.2 ± 0.89***
3	CFA+ β-sitosterol (25 mg/kg)	2 ± 0.84**	2.1 ± 0.45***	1.9 ± 0.45***	1.4 ± 0.93***
4	CFA+ β-sitosterol-SLN	1.4 ± 0.83***	1.2 ± 0.35***	0.82 ± 0.09***	0.43 ± 0.07***
5	CFA + Indomethacin (400µg/kg)	1.5 ± 0.34***	1.6 ± 0.34***	1.1 ± 0.63***	0.8 ± 0.05***

All values are presented as mean ± SEM. Statisticalanalysis by one-way ANOVA followed by Dunnett’s multiple comparison. *p < .05, **p < .01 and ***p < .01 vs. Control.

#### Effect of β-sitosterol-SLN on clinical score

**Figure 1.**
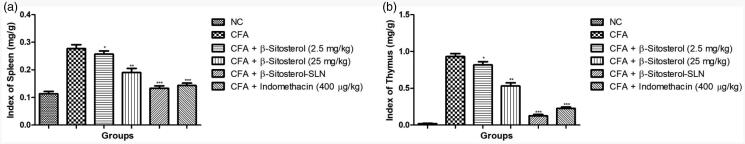
The effect of β-sitosterol-SLNs on spleen and thymus index of CFA induced rats. **a:** spleen index and **b:** thymus index. All the data presented ± SEM. Statistical analysis was performed via One-way ANOVA followed by Dennett’s test. Where *p˂.05 is significant, **p < .01 is more significant and ***p < .001 is extreme significant.

Figure 1 exhibited the clinical score of tested group rats. Normal group did not show the any sign and symptom of clinical score. CFA-induced rats demonstrated the increased clinical score, which was significantly down-regulated via β-sitosterol treatment at dose-dependent manner. Indomethacin treatment showed the reduction in the clinical score.

#### Effect of β-sitosterol-SLN on body weight

Body weight is the major factor to estimate the disease progression. Table 4 demonstrated the impact of normal and experimental rats on body weight. Normal rats showed the increased body weight from initial body weight (155.4 ± 3.92 gm) to final body weight (190.6 ± 4.98 gm). CFA induced rats exhibited the increased body weight on first 7 days (158.3 ± 4.98 gm) and after that showed the reduced body weight to every next 7 days {day 21 (155.8 ± 5.03 gm) and day 28 (151.3 ± 3.94 gm)}. j-SLN significantly (*P* < 0.001) increased the body weight {initial body weight (155.3 ± 3.45 gm) to final body weight (188.5 ± 4.08 gm)}. A similar result was obtained for the group treated with indomethacin.

**Table 4.** The effect of β-sitosterol on the body weight of CFA induced arthritic rats.

S. No	Groups	Body Weight (gm)			
		Day 7	Day 14	Day 21	Day 28
1	NC	152 ± 3.45	164.3 ± 2.98	176.4 ± 4.32	190.6 ± 4.98
2	CFA	155.4 ± 3.92	158.3 ± 4.98	155.8 ± 5.03	151.3 ± 3.94
3	CFA+ β-sitosterol (2.5 mg/kg)	154.5 ± 3.85^ns^	159.8 ± 4.11^ns^	164.5 ± 4.38*	169.5 ± 3.94**
4	CFA+ β-sitosterol (25 mg/kg)	153.9 ± 2.93^ns^	160.2 ± 3.07*	167.8 ± 3.94*	175.4 ± 4.32**
5	CFA+ β-sitosterol-SLN	155.3 ± 3.45^ns^	163.4 ± 3.94*	172.4 ± 4.34**	188.5 ± 4.08***
6	CFA + Indomethacin (400µg/kg)	154.6 ± 3.73^ns^	1.63 ± 2.93*	171.3 ± 3.45**	186.8 ± 3.84***

All values are presented as mean ± SEM. Statisticalanalysis by one-way ANOVA followed by Dunnett’s multiple comparison. *p < .05, **p < .01 and ***p < .01 vs. Control.

#### Effect of β-sitosterol-SLN on arthritic score

Arthritic score is the important parameter for the estimation of arthritis. The arthritic score of each group rat was shown in Table 5. Normal group rats showed no signs of arthritic score. CFA-induced group rats showed the arthritic score and confirm the progression of arthritis. CFA induced showed the arthritic score 4.1 ± 0.73 (day 7), 5.4 ± 0.89 (day 14), 6.9 ± 0.78 (day 21) and 7.8 ± 0.78 (day 28). β-sitosterol-SLN significantly reduced the arthritic score 3 ± 0.67 (day 7), 2.1 ± 0.54 (day 14), 1.3 ± 0.28 (day 21) and 0.6 ± 0.02 (day 28). A similar result was observed in the indomethacin group rats.

**Table 5.** The effect of β-sitosterol on the arthritic score of CFA-induced arthritic rats.

S. No	Groups	Arthritic Score			
		Day 7	Day 14	Day 21	Day 28
1	CFA	4.1 ± 0.73	5.4 ± 0.89	6.9 ± 0.78	7.8 ± 0.78
2	CFA+ β-sitosterol (2.5 mg/kg)	3.9 ± 0.78*	4.9 ± 0.78**	5.6 ± 0.83***	4.8 ± 0.78***
3	CFA+ β-sitosterol (25 mg/kg)	3.5 ± 0.78**	3.4 ± 0.63***	2.9 ± 0.67***	2.5 ± 0.73***
4	CFA+ β-sitosterol-SLN	3 ± 0.67***	2.1 ± 0.54***	1.3 ± 0.28***	0.6 ± 0.02***
5	CFA + Indomethacin (400µg/kg)	3.2 ± 0.34***	2.3 ± 0.59***	1.7 ± 0.31***	1 ± 0.09***

All values are presented as mean ± SEM. Statisticalanalysis by one-way ANOVA followed by Dunnett’s multiple comparison. *p < .05, **p < .01 and ***p < .01 vs. Control.

#### Effect of β-sitosterol-SLN on spleen and thymus index

During the arthritis, increase the thymus and spleen index is commonly observed. CFA-induced group exhibited the similar result, showed the increased thymus and spleen index. β-sitosterol (2.5 and 25 mg/kg) treated group showed the reduction in the thymus and spleen index. β-sitosterol-SLN demonstrated the maximum reduction of thymus and spleen index as compared to other treated groups (Figure 1).

#### Effect of β-sitosterol-SLN on antioxidant parameters

Table 6 showed the effect on the antioxidant parameters of experimental rats. Normal rats exhibited the TBARS (56.66 ± 2.93 nmol/g), SOD (5.43 ± 1.02 U/mg protein), CAT (5.34 ± 1.14 U/mg protein) and GSH (0.39 ± 0.04 µg/g), respectively. CFA induced rats demonstrated the increased level of TBARS (92.34 ± 4.32 nmol/g) and decreased level of SOD (1.67 ± 0.89 U/mg protein), CAT (1.76 ± 0.83 U/mg protein) and GSH (0.11 ± 0.02 µg/g). β-sitosterol-SLN significantly (*P* < 0.001) reduced the TBARS (59.65 ± 3.43 nmol/g) and increased the level of SOD (4.75 ± 1.03 U/mg protein), CAT (5.02 ± 0.87 U/mg protein) and GSH (0.34 ± 0.07 µg/g). A similar momentum was observed in the indomethacin treated group rats.

**Table 6.** The effect of β-sitosterol on the antioxidant parameter of CFA-induced arthritic rats.

S. No	Groups	Antioxidant parameters			
		TBARS (nmol/g)	SOD (U/mgprotein)	CAT (U/mgprotein)	GSH (µg/g)
1	NC	56.66 ± 2.93	5.43 ± 1.02	5.34 ± 1.14	0.39 ± 0.04
2	CFA	92.34 ± 4.32	1.67 ± 0.89	1.76 ± 0.83	0.11 ± 0.02
3	CFA+ β-sitosterol (2.5 mg/kg)	83.45 ± 2.04*	1.98 ± 0.93*	2.1 ± 0.98*	0.15 ± 0.03*
4	CFA+ β-sitosterol (25 mg/kg)	72.34 ± 2.93**	2.85 ± 0.99**	3.03 ± 0.94**	0.21 ± 0.04**
5	CFA+ β-sitosterol-SLN	59.65 ± 3.43***	4.75 ± 1.03***	5.02 ± 0.87***	0.34 ± 0.07***
6	CFA + Indomethacin (400µg/kg)	61.03 ± 2.83***	4.32 ± 1.02***	4.98 ± 0.98***	0.32 ± 0.06***

All values are presented as mean ± SEM. Statisticalanalysis by one-way ANOVA followed by Dunnett’s multiple comparison. *p < .05, **p < .01 and ***p < .01 vs. Control.

#### Effect of β-sitosterol-SLN on hematological parameters

During the arthritic disease, the level of ESR, WBC decrease and the level of Hb, RBC increase. A similar result was observed in group rats induced by CFA, and β-sitosterol-SLN altered the hematological parameters close to the normal control (Table 7). A similar result has been observed in rats of the indomethacin group.

**Table 7.** The effect of β-sitosterol on the hematological parameters of CFA-induced arthritic rats.

S. No	Groups	Hematological parameters			
		WBC	RBC	Hb	ESR
1	NC	6.45 ± 1.02	8.02 ± 1.04	13.2 ± 1.31	9.04 ± 1.02
2	CFA	8.78 ± 1.12	3.48 ± 1.11	6.75 ± 1.28	14.53 ± 1.04
3	CFA+ β-sitosterol (2.5 mg/kg)	8.34 ± 1.01*	4.02 ± 1.02**	7.14 ± 0.83*	13.89 ± 0.98**
4	CFA+ β-sitosterol (25 mg/kg)	7.78 ± 0.98**	6.03 ± 0.85***	8.98 ± 0.91***	12.45 ± 0.78***
5	CFA+ β-sitosterol-SLN	6.76 ± 0.79***	7.84 ± 1.01***	12.8 ± 1.83***	9.34 ± 0.77***
6	CFA + Indomethacin (400µg/kg)	6.97 ± 0.83***	7.67 ± 0.89***	12.67 ± 1.92***	9.45 ± 0.69***

All values are presented as mean ± SEM. Statisticalanalysis by one-way ANOVA followed by Dunnett’s multiple comparison. *p < .05, **p < .01 and ***p < .01 vs. Control.

#### Effect of β-sitosterol-SLN on hepatic parameters

Table 8 exhibited the effect of normal and experimental rats on the hepatic parameters. Hepatic parameters such as AST (40.3 ± 2.34 U/L), ALT (45.3 ± 1.03 U/L) and ALP (78.5 ± 2.34 U/L) was found in the normal rats and CFA induced rats showed the increased level of AST (142.3 ± 3.45 U/L), ALT (170.3 ± 2.12 U/L) and ALP (411.3 ± 6.56 U/L), respectively. β-sitosterol-SLN significantly (*P* < 0.001) reduced the level of AST (50.4 ± 1.89 U/L), ALT (60.3 ± 2.92 U/L) and ALP (100.3 ± 3.84 U/L) and almost similar result was observed in the indomethacin group rats.

**Table 8.** The effect of β-sitosterol on the hepatic parameters of CFA-induced arthritic rats.

S. No	Groups	Hepatic parameters		
		AST (U/L)	ALT (U/L)	ALP (U/L)
1	NC	40.3 ± 2.34	45.3 ± 1.03	78.5 ± 2.34
2	CFA	142.3 ± 3.45	170.3 ± 2.12	411.3 ± 6.56
3	CFA+ β-sitosterol (2.5 mg/kg)	132.3 ± 4.23*	155.6 ± 3.12**	350.3 ± 4.95**
4	CFA+ β-sitosterol (25 mg/kg)	100 ± 2.34**	111.3 ± 3.84***	280.4 ± 3.89***
5	CFA+ β-sitosterol-SLN	50.4 ± 1.89***	60.3 ± 2.92***	100.3 ± 3.84***
6	CFA + Indomethacin (400µg/kg)	55.8 ± 2.93***	69.4 ± 3.01***	111.5 ± 4.56***

All values are presented as mean ± SEM. Statisticalanalysis by one-way ANOVA followed by Dunnett’s multiple comparison. *p < .05, **p < .01 and ***p < .01 vs. Control.

#### Effect of β-sitosterol-SLN on cytokines

**Figure 2.**
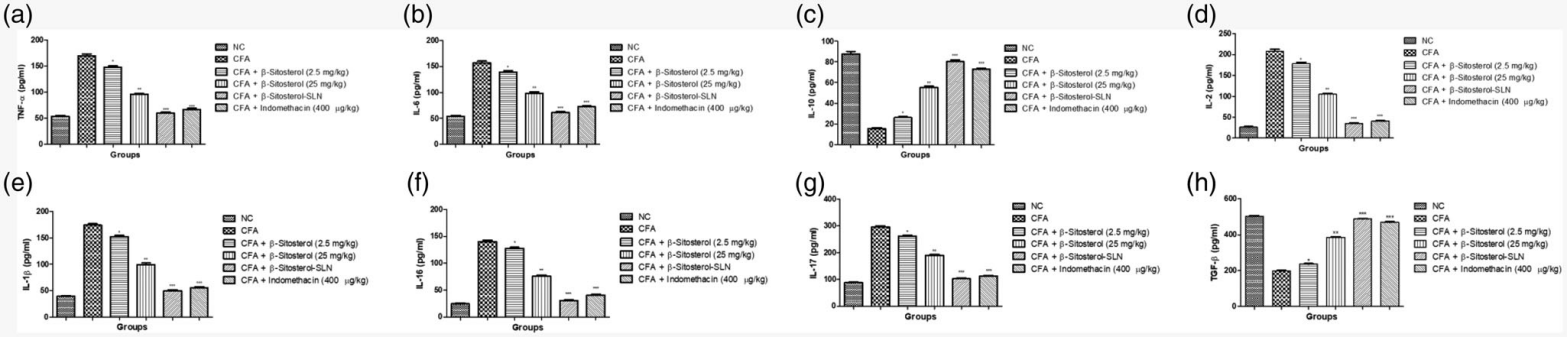
The effect of β-sitosterol-SLNs on cytokines level (Serum) of CFA induced rats. **a:** TNF-α, **b:** Il-6, **c:** IL-10, **d:** IL-2, **e:** IL-1β, **f:** IL-16, **g:** IL-17 and **h:** TGF-β. All the data presented ± SEM. Statistical analysis was performed via One-way ANOVA followed by Dennett’s test. Where *p˂ .05 is significant, **p < .01 is more significant and ***p < .001 is extreme significant.

Figure 2 showed the effect of the β-sitosterol-SLN on cytokine levels of experimental rats serum. CFA induced rats showed the increased level of TNF-α (170.46 ± 8.46 pg/ml), IL-1β (175.67 ± 6.53 pg/ml), IL-2 (208.04 ± 5.84 pg/ml), IL-6 (156.54 ± 4.34 pg/ml), IL-16 (140.45 ± 5.94 pg/ml), IL-17 (297.45 ± 6.35 pg/ml) and reduced the level of IL-10 (15.43 ± 0.56 pg/ml), TGF-β (197.45 ± 6.54 pg/ml). β-sitosterol-SLN significantly (*p* < .001) reduced the TNF-α (60.42 ± 2.35 pg/ml), IL-1β (50.43 ± 2.04 pg/ml), IL-6 (61.04 ± 2.34 pg/ml), IL-2 (34.05 ± 1.83 pg/ml), IL-16 (30.5 ± 2.38 pg/ml), IL-1β (18.54 ± 3.94 pg/ml), IL-17 (102.5 ± 3.82 pg/ml) and increased the IL-10 (80.45 ± 2.80 pg/ml), TGF-β (487.94 ± 10.45 pg/ml) as compared to CFA and other treated group.

**Figure 3.**
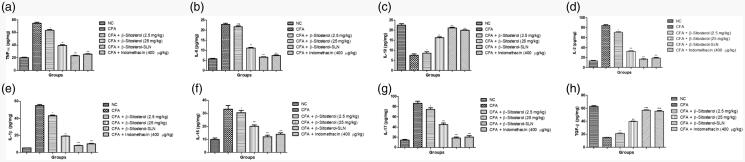
The effect of β-sitosterol-SLNs on cytokines level (tissue) of CFA induced rats. **a:** TNF-α, **b:** Il-6, **c:** IL-10, **d:** IL-2, **e:** IL-1β, **f:** IL-16, **g:** IL-17 and **h:** TGF-β. All the data presented ± SEM. Statistical analysis was performed via One-way ANOVA followed by Dennett’s test. Where *p˂ .05 is significant, **p < .01 is more significant and ***p < .001 is extreme significant.

Figure 3 showed the effect of the β-sitosterol-SLN on cytokine levels of experimental rats tissue. CFA induced rats showed the increased level of TNF-α (74.95 ± 2.34 pg/mg), IL-1β (55.5 ± 2.04 pg/mg), IL-2 (84.5 ± 2.94 pg/mg), IL-6 (22.43 ± 1.93 pg/mg), IL-16 (33.43 ± 2.09 pg/mg), IL-17 (86.85 ± 3.05 pg/mg) and reduced the IL-10 (7.5 ± 0.94 pg/mg), TGF-β (15.2 ± 1.47 pg/mg) as compared to normal rats and β-sitosterol-SLN significantly (*p* <.001) reduced the TNF-α (23.41 ± 1.97 pg/mg), IL-1β (8.12 ± 0.74 pg/mg), IL-6 (12.03 ± 0.91 pg/mg), IL-2 (16.5 ± 1.84 pg/mg), IL-16 (12.3 ± 1.93 pg/mg), IL-1β (18.54 ± 3.94 pg/mg), IL-17 (17.34 ± 2.04 pg/mg) and increased the IL-10 (21.43 ± 1.82 pg/mg), TGF-β (57.65 ± 3.46 pg/mg) as compared to CFA control rats.

#### Effect of β-sitosterol-SLN on inflammatory mediators

**Figure 4.**
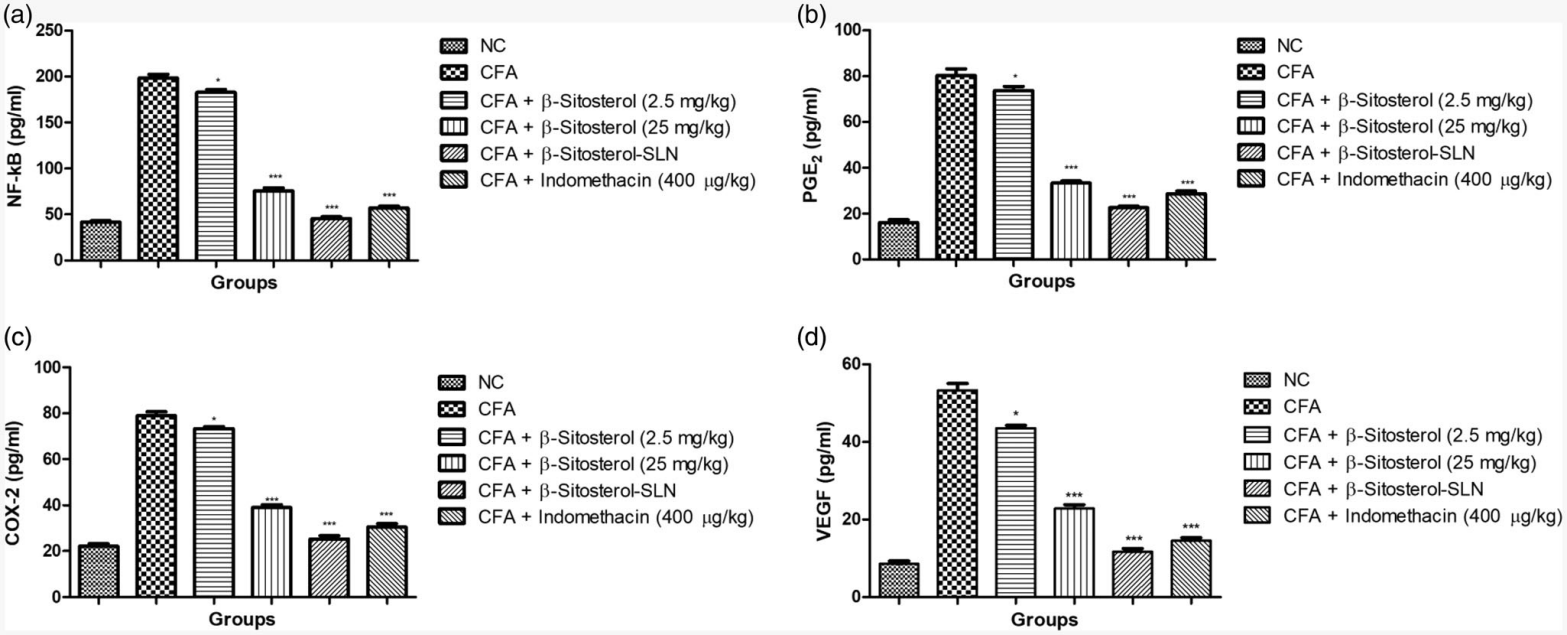
The effect of β-sitosterol-SLNs on inflammatory parameter (Serum) of CFA induced rats. **a:** NF-κB, **b:** PGE_2_, **c:** COX-2 and **d:** VEGF. All the data presented ± SEM. Statistical analysis was performed via One-way ANOVA followed by Dennett’s test. Where *p˂.05 is significant, **p < .01 is more significant and ***p < .001 is extreme significant.

Figure 4 demonstrated the effect of β-sitosterol-SLN on the inflammatory mediator of experimental rats serum. CFA-induced rats showed the increased level of NF-κB (198.34 ± 4.56 pg/ml), PGE_2_ (80.64 ± 2.91 pg/ml), COX-2 (79.03 ± 3.84 pg/ml), VEGF (53.95 ± 2.90 pg/ml) as compared to control group rats. β-sitosterol-SLN significantly (*p* < .001) decreased the level of NF-κB (45.67 ± 2.05 pg/ml), PGE_2_ (22.41 ± 1.03 pg/ml), COX-2 (25.41 ± 1.48 pg/ml), VEGF (11.68 ± 1.14 pg/ml) as compared to CFA control group rats.

**Figure 5.**
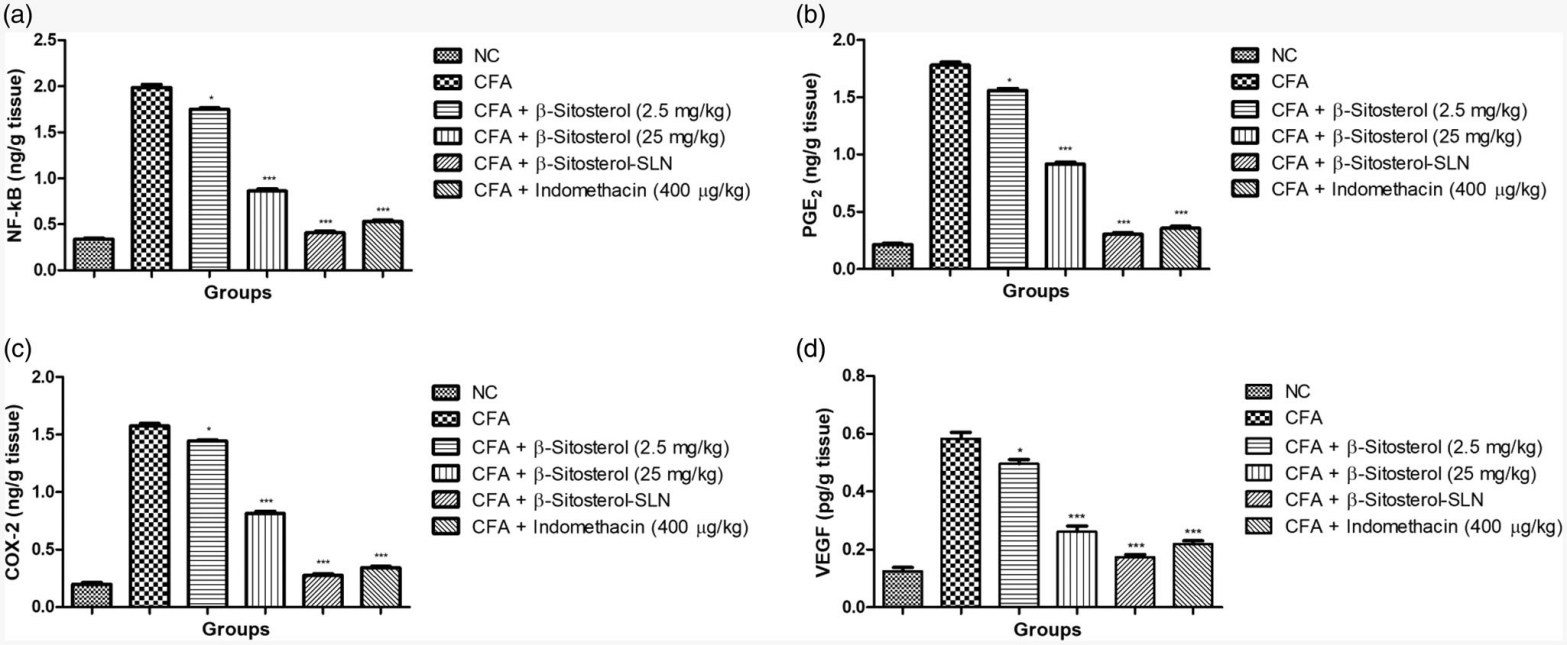
The effect of β-sitosterol-SLNs on inflammatory parameter (tissue) of CFA induced rats. **a:** NF-κB, **b:** PGE_2_, **c:** COX-2 and **d:** VEGF. All the data presented ± SEM. Statistical analysis was performed via One-way ANOVA followed by Dennett’s test. Where *p˂.05 is significant, **p < .01 is more significant and *** *p* < .001 is extreme significant.

Figure 5 demonstrated the effect of β-sitosterol-SLN on the inflammatory mediator of experimental rats serum. CFA induced rats showed the increased level of NF-κB (1.98 ± 0.58 pg/mg), PGE_2_ (1.78 ± 0.74 pg/mg), COX-2 (1.76 ± 0.71 pg/mg), VEGF (0.58 ± 0.15 pg/mg) as compared to control group rats. β-sitosterol-SLN significantly (*p* < .001) decreased the level of NF-κB (0.42 ± 0.12 pg/mg), PGE_2_ (0.31 ± 0.09 pg/mg), COX-2 (0.28 ± 0.3 pg/mg), VEGF (0.18 ± 0.03 pg/mg) as compared to CFA control group rats.

#### Effect of β-sitosterol-SLN on HIF1α and ANG-1

**Figure 6.**
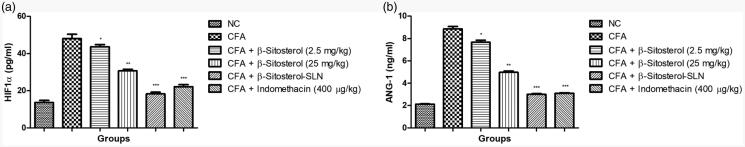
The effect of β-sitosterol-SLNs on HIF1α and ANG-1 parameter of CFA induced rats. **a:** HIF1α and **b:** ANG-1. All the data presented ± SEM. Statistical analysis was performed via One-way ANOVA followed by Dennett’s test. Where *p˂.05 is significant, **p < .01 is more significant and ***p < .001 is extreme significant.

During the arthritis, boosted the level of HIF1α and ANG-1, a similar result was obtained in the CFA group rats. β-sitosterol-SLN significantly (*P* < 0.001) reduced the level of HIF1α and ANG-1 as compared to other groups. β-sitosterol (2.5 and 25 mg/kg) and indomethacin treated group rats showed the significantly suppression of the level of HIF1α and ANG-1 (Figure 6).

### Quantitative RT-PCR gene expression

**Figure 7.**
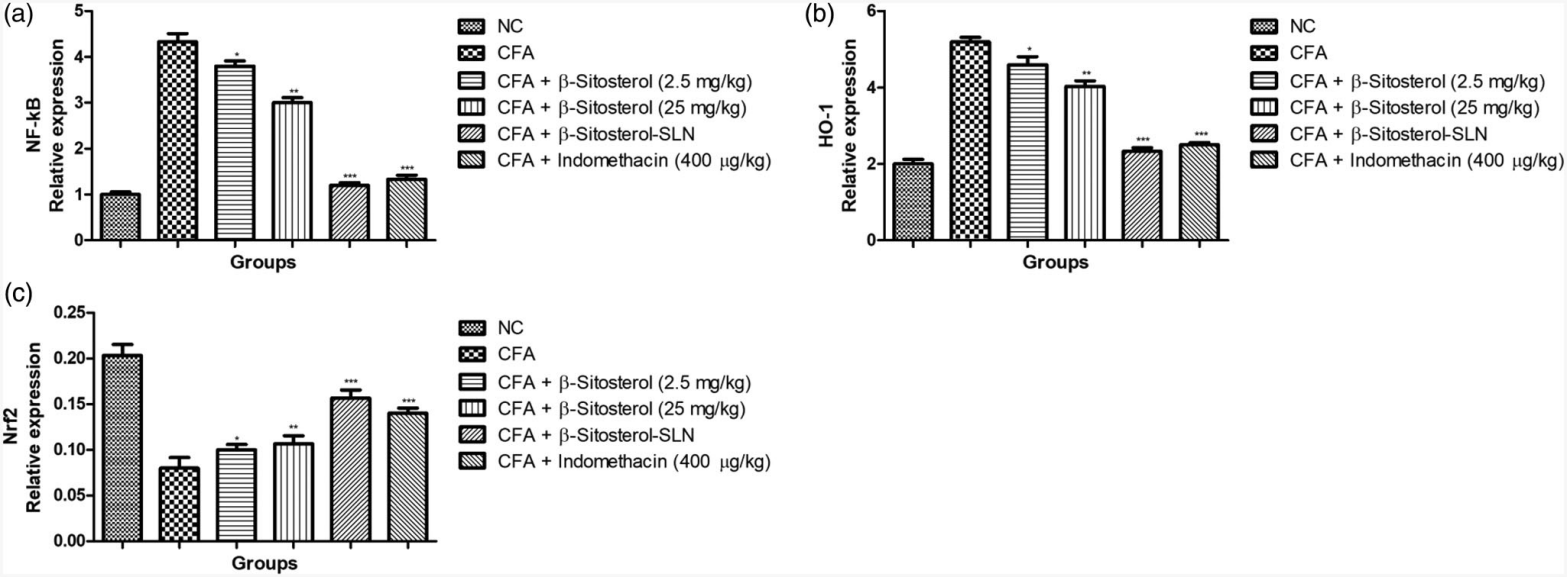
The effect of β-sitosterol-SLNs on mRNA expression (HO-1, Nrf_2_ and NF-κB) of CFA induced rats. **a:** HO-1, **b:** Nrf_2_ and **c:** NF-κB. All the data presented ± SEM. Statistical analysis was performed via One-way ANOVA followed by Dennett’s test. Where *p˂.05 is significant, **p < .01 is more significant and ***p < .001 is extreme significant.

Figure 7 showed the effect of β-sitosterol-SLN on the different gene expression. CFA induced rats showed the increased expression of NF-κB, HO-1 and reduced expression of Nrf2. β-sitosterol-SLN significantly (*p* < .001) suppressed the expression of NF-κB, HO-1 and boosted the expression of HO-1.

**Figure 8.**
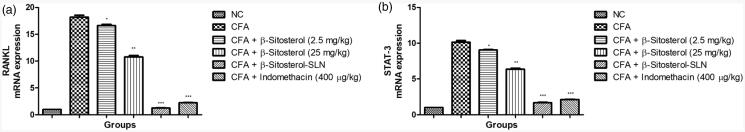
The effect of β-sitosterol-SLNs on RANKL and STAT-3 expression of CFA induced rats. **a:** RANKL and **b:** STAT-3. All the data presented ± SEM. Statistical analysis was performed via One-way ANOVA followed by Dennett’s test. Where *p˂.05 is significant, **p < .01 is more significant and ***p < .001 is extreme significant.

Figure 8 demonstrated the expression of RANKL and STAT-3. CFA induced rats showed the increased expression of RANKL and STAT-3. β-sitosterol-SLN significantly (*p* < .001) reduced the expression of RANKL and STAT-3.

## Discussion

SLNs provide a novel deliver physicochemically compromised and poorly permeable drug across the gastrointestinal (GI) mucosa, resulting considerably enhanced the plasma concentration (Arora et al., 2015). Nano-sized and subsequently enhanced the surface area of nanoparticles may show the high bio-adhesion to the GI wall, ensuring the higher, effective and prolonged uptake β-sitosterol embedded in the SLN's solid lipid matrix is expected to be protected against enzymatic degradation, both in the gut and through the liver. Surfactant and co-surfactant used in the preparation of β-sitosterol-SLNs contributed to their increased permeability across the intestinal membrane. Although free β-sitosterol undergoes for first pass metabolism, SLNs are allegedly lymphatically absorbed which further bypass the 1st pass circulation via liver for orally administration (Arora et al., 2015). Particle of size < 200 trend to bypass reticuloendothelial system pickup via spleen and liver, thereby avoiding liver (major site of drug degradation and metabolism of β-sitosterol). Thus, β-sitosterol-SLNs (146.7 nm) will remain in circulation for prolonged periods, achieve prolonged high blood levels and decrease clearance after absorption. Therefore, enhanced systemic bioavailability complemented by enhanced penetrability as shown by their preferential distribution into various tissues such as bones (Thakkar et al., 2007) may obvious itself in achieving higher concentration in the joint synovium, showing higher anti-inflammatory response in CFA-inflamed joints.

Rheumatoid, symptom like deformity, swelling in the ankle or joint, systemic change and release of autoantibody is an autoimmune disease categorized via generation of chronic inflammation in the synovial joint (Kumar et al., 2015a, 2016b). During RA condition, expansion of swelling of the synovium due to the proliferation of synovial cells and take part in the cartilage deterioration. Prolonged inflammation during the RA, linked with the bone erosion and affected 80% of patients and occurs rapidly. Additionally, the misbalance in the immune functions especially reflects the imbalance between the humoral immunity and cellular immunity. Research suggest that the cellular immunity activated the T1 cells, thus start the secretion of pro-inflammatory cytokines and humoral immunity reduced the secretion of T2 inflammatory cytokines (Rahman et al., 2017). Previous studies suggest that the production of cytokines start in the lymphocytes in the synovium and mononuclear macrophages and play a crucial role in the pathogenesis of RA. Though cytokines have a critical role in the persistence of RA disease (Kumar et al., 2015a; Rahman et al., 2015, 2017).

The CFA-induced arthritis model is commonly used to analyze the anti-arthritis potential of the drugs tested. Inspired by the clarification of β-sitosterol-SLN's anti-inflammatory ability and to explore its long-term anti-inflammatory effects (Kumar et al., 2015a, c). In this experimental study, the β-sitosterol-SLN was then evaluated against arthritis induced by the adjuvant-induced immunological chronic inflammation, especially complete Fruend adjuvant (CFA). That causes common human arthritis and is more common to pathological and clinical circumstances. Previous work indicates that adjuvant initiates the secretion of inflammatory mediators and stimulates irritated phagocytes that play a major role in fibrosis, vascular and tissue degradation over time (Kumar et al., 2015a, c). After injecting the adjuvant into the rat, in the first phase start the swelling into the hind paw and progressively increased (which continued for next few weeks) and after the few week, adjuvant start the accumulation into the soft tissue of rat and expand the nodules in tail, ear contralateral part and front paw of experimental rats (Kumar et al., 2015a, c, 2016b). β-sitosterol-SLN significantly reduced the humoral immune reaction due to suppression the acute inflammation via down-regulating the vascular permeability and inhibiting the pro-inflammatory cytokines. Adjuvant develops the secondary reaction after the 2 weeks and significantly reduced by β-sitosterol-SLN. Apparently, CFA-induced secondary lesions were exposed to delayed hypersensitivity reactions and β-sitosterol-SLN also had an apparent effect on this, showing its antiarthritic effect.

Arthritic rats induced by CFA demonstrated the reduced body weight until the end of the experimental test (28 days). Previous research indicates that the decreased body weight observed in CFA mediated group rats due to a lack of leucine and glucose absorption by the intestines (Kumar et al., 2015a, c, 2016b, a). β-sitosterol-SLN significantly (*p* < .001) increased the body weight via increase the absorption of leucine and glucose via intestine.

Previous research suggests that during the arthritic condition, the level of Hb, RBC was reduced and WBC, ECR level was increased. Arthritic condition, the level of RBC reduced and produced the animatic condition due to erythrocyte deformability (shorten life span of erythrocytes) (Aiyalu et al., 2016; Mahdi et al., 2018). Another hematological parameter such as Hb, reduced observed during the arthritic condition, resultant suppression of bone marrow erythropoietin and destruction of premature RBCs (Patil et al., 2011; Premaratna et al., 2011). β-sitosterol-SLN significantly (*P* < 0.001) restored the level of RBC and Hb near the normal level and support the anti-arthritic effect of β-sitosterol-SLN. WBC is an important component of the immune system linked to the induction of an inflammatory reaction and its associated other diseases (Aiyalu et al., 2016; Kumar et al., 2016b). Clinical study suggests that IL-1β increased the level of WBC and start the production of inflammatory component like granulocyte and macrophages and also induce the growth of colony stimulating factors (Kim and Park 2006). CFA-induced arthritic rats treated with β-sitosterol-SLN significantly (*p* < 0.001) reduced the migration of inflammatory granulocytes and macrophage. Another hematological component, ESR, begins the production of endogenous proteins such as α/β globulin and fibrinogen and suggests arthritic disease progression (Kumar et al., 2016b).

Research suggests that reactive oxygen species (ROS)/reactive nitrogen species (RNS) are generated in normal cellular metabolism inside the body and play an important role in attacking the microbial agent. Any disparity between ROS/RNS production and inactivation leads to cellular dysfunction and unwanted condition of inflammatory diseases such as arthritis. As we know, that these ROS having the damaging property, body has developed numerous antioxidant mechanism to prevent the ROS induced damage. During the arthritic condition, enhanced the ROS synthesis via activated the neutrophils and phagocyte during the oxidative rush, which cross the limit of endogenous antioxidant resultant induce the oxidative stress. Continuous production or generation of ROS in the synovial membrane initiates damaging nucleic acid, collagen, lipids and protein, which additionally turns into signals for the inflammatory cells and deteriorates the condition of arthritic disease. Research suggests that ROS acts as a stimulating signal to activate the NF-kB pathway, leading to alterations of different transcription mediators and pro-inflammatory cytokines. In the current experimental study, immunization with adjuvant resultant in the suppression the level of endogenous antioxidant like GSH, CAT and SOD and enhancement of pro-oxidant like TBARS in arthritic rats. β-sitosterol-SLN significantly (*P* < 0.001) increased the level of endogenous antioxidant like GSH, CAT and SOD and reduced the level of pro-oxidant like TBARS as compared to arthritic rats.

Previous research suggests that during the RA condition, the cytokines divided into 2 groups like one group produced via lymphocytes and secrete the macrophages/monocytes viz., TNF-α, IL-1β, IL-6, IL-10, IL-16, IL-17 and IL-18. Previous research also suggests that the various factors directly or indirectly interact with the cytokines, which further constrain or boost the incidence and expansion of numerous disease. TNF-α is a well-known cytokines that affect the various functions implicated in the RA pathogenesis such as accumulating leukocytes, chemokines, activation chondrocytes and osteoclasts, activated endothelial cells, endorsing articular dysfunction and nociceptor sensitization (Brennan & McInnes, 2008). TNF-α is the present high amount during the RA disease and its induce the local joint tissue destruction and other clinical symptoms. According to Kumar et al., the high amount of TNF-α was found in the RA rats and targeting the TNF-α for relief the RA disease (Kumar et al., 2015a). Previous research suggests that the IL-1β is the essential factor for the expansion of RA pathology and present in the joint cavity and it can support the cell migration and enhance the endothelial cells. Targeting the IL-1β is best way to treat RA disease. Another pro-inflammatory cytokine like IL-16 released from the T lymphocytes and expand the RA disease. During the RA, IL-16 not only showed the effect on the cartilage collagen but also reduces the bone synthesis and boosts the osteoclast differentiation. Another pro-inflammatory cytokine such as IL-6 play a synergetic role with IL-1β and TNF-α to increase the inflammatory reactions. In the current experimental study, we scrutiny the role of inflammatory cytokines and lymphocytes in CFA-induced arthritis and explore the mechanism at cellular and molecular levels. β-sitosterol-SLN significantly (*p* < .001) reduced the inflammatory response in the synovial cavity in the CFA-induced RA rats and decrease the arthritic grading of synovitis via reducing the level of IL-1β, IL-6, IL-16 and TNF-α in the serum of experimental rats.

NF-κB plays an important role in the expansion of arthritis and various researchers targeting the NF-κB signaling pathway to treat the arthritic disease. NF-κB plays a role in regulating/circulating the immune-inflammatory response (Brennan & McInnes, 2008; Isomäki, 2012). NF-κB increases the specific genes such as chemokines, cytokines and histocompatibility complex and receptors that are necessary for the immune cell migration and adhesion in addition to apoptosis and cellular proliferation. In the synovial biopsy of animal and human exhibited the over-expression of NF-κB (Kamel et al., 2018). In this study, CFA treated rats showed the increased level of NF-κB and β-sitosterol-SLN significantly (*P* < 0.001) decreased the NF-κB level in the serum and tissue. β-sitosterol-SLN reduced the level of NF-κB might be due to suppression of significant pro-inflammatory cytokines IL-17. In support, Kim et al., claimed that β-sitosterol showed the anti-inflammatory effect in high fat diet induced intestinal inflammation via suppression of NF-κB pathway (Kim et al., 2014).

Previous research suggest that the Nrf_2_ is a significant transcription factor commonly present to induce the numerous set of antioxidant enzymes like HO-1, GPx and glutathione S-transferanse, via binding a sequence of DNA called anti-oxidant response elements (ARE), which reduced the pro-inflammatory pathway and oxidative stress. Previous animal investigation suggests the potential relation between the NF-kB and Nrf_2_, and showed the joint destruction and oxidative stress in rats. CFA induced rats exhibited the increased expression of HO-1 and Nrf2 and β-sitosterol-SLN significantly (*P* < 0.001) downregulated the expression of HO-1 and Nrf2. On the basis of result, we can conclude that β-sitosterol-SLN reduced the pro-inflammatory cytokines and oxidative stress due to modulating the HO-1/Nrf2 signaling pathway.

It is well documented that STAT-3 play an important role in the proliferation during the RA. STAT-3 is a prime element for TH17 lymphocyte proliferation and fibroblast like synoviocyte in RA (Krause et al., 2002). CFA treated rats showed the increased expression of STAT-3 and β-sitosterol significantly reduced the expression of STAT-3. Previous study suggest that the STAT-3 activate through IL-6 cytokines and suppressive effect of β-sitosterol-SLN might be due to anti-inflammatory effect (Zhou et al., 2007). According to the Kamel et al., STAT-3 is consider as the excellent target for gene therapy during the RA reported that suppressing STAT-3 funtion induces the synoviocyte apoptosis and converts the endogenous growth factors include epidermal growth factor into death factors (Kamel et al., 2018).

Angiogenesis, an exciting proliferation of the blood vessels, continually delivers immune cells (chemotaxis) and cytokines for arthritic lesion development. Angiogenesis, play an important role in the maintenance and expansion of RA pannus (Paleolog, 1996). VEGF (angiogenic growth factor) is a significant mediator responsible for the changes at the affected joints via providing oxygen and nutrients essential for maintaining the proliferation, synoviocyte proliferation, enhance vascular permeability and persistent nature of arthritic pannus which may start the swelling in the joint (Paleolog & Miotla, 1998; Bainbridge et al., 2006). Previous studies suggest that the various factors such as IL-17, TNF-α, Il-6 and ROS are capable for induction the VEGF (Kamel et al., 2018). Β-sitosterol-SLN significantly inhibited the VEGF level in the serum and tissue might be due to its anti-inflammatory and antioxidant effect. In support, it was showed that β-sitosterol is an effectual VEGF suppressor in rat model of renal carcinogenesis (Sharmila & Sindhu 2017).

## Conclusion

The current investigation showed the development of optimized β-sitosterol-SLNs which showed the satisfactory loading capability, entrapment efficiency, particle size distribution and *invitro* release characteristics. β-sitosterol-SLNs exerted anti-arthritic effect probably due to its anti-inflammatory and anti-oxidant effect. It is plausible due to directly related to the reduction the cytokines and inflammatory mediators, suppression the fundamental gene such as STAT-3 as well as scavenges ROS.

## Supplementary Material

Supplemental Material

Supplemental Material
