# Supplementary figures and images for: β-Sitosterol-loaded solid lipid nanoparticles ameliorate complete Freund’s adjuvant-induced arthritis in rats: involvement of NF-кB and HO-1/Nrf-2 pathway
Source: Drug Deliv. 2020 Sep 18;27(1):1329–41. doi: 10.1080/10717544.2020.1818883 (PMC7534215; doi:10.1080/10717544.2020.1818883)

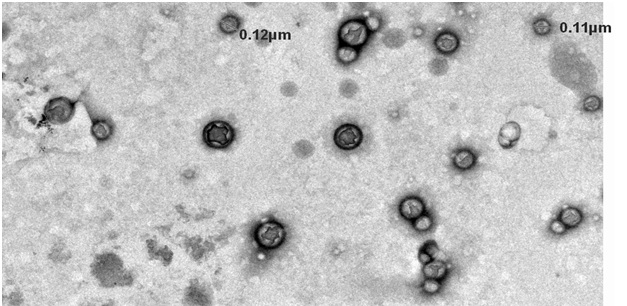


**Supplementary figure 1:** showed the TEM image of β-Sitosterol-SLNs

Supplement: Supplemental Material [file IDRD_A_1818883_SM8057.docx]
