# Supplementary material for: β-Sitosterol-loaded solid lipid nanoparticles ameliorate complete Freund’s adjuvant-induced arthritis in rats: involvement of NF-кB and HO-1/Nrf-2 pathway
Source: Drug Deliv. 2020 Sep 18;27(1):1329–41. doi: 10.1080/10717544.2020.1818883 (PMC7534215; doi:10.1080/10717544.2020.1818883)
Supplement: Supplemental Material [file IDRD_A_1818883_SM8055.docx]

**Supplementary table 1:** Particle size, entrapment efficiency and loading capacity of SLN after storage at 25^◦^C/60%RH

| **Time (in weeks)** | **Particle size (nm)** | **PDI** | **EE (%)** | **LC (%)** |
| --- | --- | --- | --- | --- |
| 0 | 73.06 | 0.152 | 90 | 14.1 |
| 1 | 70 | 0.153 | 89.2 | 13.7 |
| 2 | 69.2 | 0. 23 | 88.1 | 13.2 |
| 4 | 68.3 | 0. 26 | 87.3 | 12.8 |
| 8 | 67.6 | 0.31 | 87.1 | 12.5 |
| 12 | 67 | 0. 34 | 86.8 | 12.3 |

SLN: Solid lipid nanoparticles; EE: Entrapment Efficiency; LC: Loading Capacity; PDI: Polydispersity Index;

**Supplementary table 2:** Particle size, entrapment efficiency and loading capacity of SLN after storage at 40^◦^C/75%RH.

| **Time (in weeks)** | **Particle size (nm)** | **PDI** | **EE (%)** | **LC (%)** |
| --- | --- | --- | --- | --- |
| 0 | 66.01 | 0.143 | 90 | 14.2 |
| 1 | 69.16 | 0.148 | 88.8 | 13.6 |
| 2 | 73.32 | 0.22 | 84.5 | 13.1 |
| 4 | 80.14 | 0.35 | 81.12 | 12.8 |
| 8 | 98.35 | 0.48 | 78.34 | 12.1 |
| 12 | 105 | 0.55 | 74.54 | 11.6 |
